# An automatic cough counting method and system construction for portable devices

**DOI:** 10.3389/fbioe.2024.1477694

**Published:** 2024-09-27

**Authors:** Yixuan Wang, Kehaoyu Yang, Shaofeng Xu, Shuwang Rui, Jiaxing Xie, Juncheng Wang, Xin Wang

**Affiliations:** ^1^ Engineering Training Centre, Beihang University, Beijing, China; ^2^ School of Automation Science and Electrical Engineering, Beihang University, Beijing, China; ^3^ Liupanshan Laboratory, Yinchuan, China; ^4^ State Key Laboratory of Respiratory Disease, Guangzhou, China; ^5^ Institute of Stomatology, First Medical Center, Chinese PLA General Hospital, Beijing, China; ^6^ Nineth Medical Center of PLA General Hospital Gynaecology and Obstetrics, Chinese PLA General Hospital, Beijing, China

**Keywords:** cough counting, support vector machine, Mel frequency cepstrum coefficient, short-time feature, portable devices

## Abstract

**Introduction:**

Cough is a common symptom of respiratory diseases, and prolonged monitoring of cough can help assist doctors in making judgments about patients’ conditions, among which cough frequency is an indicator that characterizes the state of the patient’s lungs. Therefore, the aim of this paper is to design an automatic cough counting system to monitor the number of coughs per minute for a long period of time.

**Methods:**

In this paper, a complete cough counting process is proposed, including denoising, segment extraction, eigenvalue calculation, recognition, and counting process; and a wearable automatic cough counting device containing acquisition and reception software. The design and construction of the algorithm is based on realistically captured cough-containing audio from 50 patients, combined with short-time features, and Meier cepstrum coefficients as features characterizing the cough.

**Results:**

The accuracy, sensitivity, specificity, and F1 score of the method were 93.24%, 97.58%, 86.97%, and 94.47%, respectively, with a Kappa value of 0.9209, an average counting error of 0.46 counts for a 60-s speech segment, and an average runtime of 2.80 ± 2.27 s.

**Discussion:**

This method improves the double threshold method in terms of the threshold and eigenvalues of the cough segments’ sensitivity and has better performance in terms of accuracy, real-time performance, and computing speed, which can be applied to real-time cough counting and monitoring in small portable devices with limited computing power. The developed wearable portable automatic cough counting device and the accompanying host computer software application can realize the long-term monitoring of patients’ coughing condition.

## 1 Introduction

Cough is a common symptom of respiratory diseases, which is caused by inflammation, foreign bodies, chemical or physical stimulation of trachea, bronchial mucosa, or pleura. It is a protective reaction for the human body, which can help discharge the foreign body in the airway and remove the secretion. However, if the cough continues and turns to chronic cough from acute cough, it often brings great pain to the patient, such as chest tightness, pharyngeal itching, and wheezing.

Recent years, medical academic groups such as the British Thoracic Society, the American College of Chest Physicians, the European Respiratory Society, the Japanese Respiratory Society, and the French Society of Otolaryngology have issued guidelines for cough diagnosis and treatment, acknowledging the importance of cough diagnosis. However, most of them focus on the subjective evaluation of cough, that is, through the cough visual analog scale (VAS), cough severity index (CSI), cough questionnaire, and other tools to evaluate the patient’s cough condition (Cho et al., 2019; Birring and Spinou, 2015).

The cough sound detection algorithm mainly focuses on the feature extraction and recognition of cough sounds (Ijaz et al., 2022; Serrurier et al., 2022). In the aspect of feature extraction, researchers mainly calculate acoustic features of cough sounds. Mel frequency cepstrum coefficients (Sun et al., 2011; Larson et al., 2012; Drugman et al., 2013; Miranda et al., 2019; Barata et al., 2023) and short-time energy parameters (Drugman et al., 2013; Drugman et al., 2020; Monge-Alvarez et al., 2019) are used as eigenvalues to reflect the information of cough sound signals. Other features such as spectrogram (Amoh and Odame, 2016; Cesnakova et al., 2019), non-negative matrix factorization (You et al., 2017), spectral entropy, and Hu moment (den Brinker et al., 2021) also have a small number of applications. It is common to use the neural network (Miranda et al., 2019; Cesnakova et al., 2019; Barry et al., 2006), support vector machine (SVM) (Liu et al., 2013; Bhateja et al., 2019), and hidden Markov model (Shin et al., 2009; Drugman et al., 2012) for cough sound recognition or classification.

At present, the automatic cough recognition and counting system mainly includes Leicester Cough Monitor (LCM), VitaloJAK, and LEOSound, and LCM is widely used. Based on MFCC parameters and hidden Markov model, the system realizes 24-h continuous dynamic cough recording (Matos et al., 2007; Birring et al., 2008; Kulnik et al., 2016). VitaloJAK is composed of a microphone and a recording device, which can evaluate the cough frequency of patients objectively (McGuinness et al., 2012). LEOSound is a portable mobile system for automatic long-term recording and analyzing respiratory sounds. It realizes long-term lung sound recording (including cough, wheezing, etc.) through a bioacoustic sensor attached to the human body (Koehler et al., 2014; Krönig et al., 2017). Such systems can automatically identify and record cough frequency, but the correlation between the results and the subjective cough severity is weak and still needs to be combined with other cough assessment methods. Shin et al. (2009) designed a HMM hybrid model using ANN-improved MFCC parameters to identify cough sounds and realize automatic detection of the abnormal health status of the elderly. Amrulloh et al. (2015) developed a method for automatic identification of cough segments from children’s recordings. Non-Gaussianity, Shannon entropy, and cepstrum coefficients were extracted as characteristic parameters to describe cough, and the artificial neural network was trained to realize automatic counting of children’s cough. Peng et al. (2023) designed a lightweight cough detection system based on CNN, using the hardware and software co-design method; developed a dedicated hardware accelerator to perform calculations efficiently; and achieved lower complexity and power consumption. Pahar et al. (2023) proposed a bed occupancy detection system based on LSTM, which combines the signals obtained by the three-axis accelerometer to perform automatic long-term cough monitoring.

Due to the lack of public cough speech data with sufficient samples and no recognized gold standard, although the judgment methods of cough fragments emerge in an endless stream, it is still impossible to objectively evaluate the detection effect. Most of the researchers focus on the distinction between cough and non-cough segments or use computer processors as a computing tool for the system, which lacks the method construction in the actual application environment, and thus unable to achieve real-time monitoring; however, the detection system based on the actual environment lacks high accuracy, and the detection effect is unsatisfactory. In addition, with the rapid development of voice analysis technology, accurate cough detection systems will be widely used in home care and clinical trials in the near future.

Cough frequency represents the number of coughs over a period of time, which can be used as a discriminant index for long-term monitoring of lung status in patients with respiratory diseases. It has an important reference value in the early stage of the disease (Hall et al., 2020; Turner et al., 2018; Yousaf et al., 2013) and enables monitoring of respiratory diseases such as COPD in a non-invasive manner. Based on the above background, an improved double-threshold cough tone counting method is proposed, and a wearable portable automatic cough counting device with accompanying host computer software is designed and developed in this article. The main contributions of this article are as follows:

(1) An improved dual-threshold cough tone counting method considering the eigenvalue of extra threshold is proposed to extract potential cough segments from the audio, which improved the sensitivity, the computational speed, and balanced real-time and accuracy.
2) A set of wearable automatic cough counting device is developed to realize long time monitoring of patient’s cough, which improved the accuracy in low-power devices.

The rest of this work is organized as follows: the data-obtaining method, process method, and system design are proposed in Section 2. The processing results are given in Section 3. Section 4 presents the discussion. Section 5 presents the conclusion and future work.

## 2 Materials and methods

### 2.1 Methods and process of obtaining raw data

All the data used in this paper were collected from clinical patients in Guangdong Medical University, including 10–45 min of audio recorded by 50 patients in their daily state. The audio data used in the trial were obtained by placing the Philips Sound Recorder VTR5000 at the patient’s bedside, recording the patient’s mouth sounds, and assessing the quality of the audio when collection was complete, removing audio that had no coughing sound or was extremely unclear.

### 2.2 Specific methods

**FIGURE 1:**
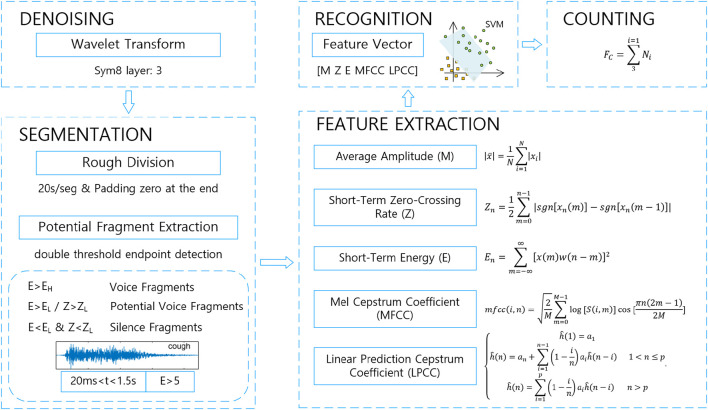
Algorithm flow chart.

The corresponding processing algorithm is designed according to the collected cough sound data above, including signal denoising, rough division and double threshold endpoint detection, feature extraction, cough sound recognition, and cough counting process per minute, which is shown in Figure 1.

#### 2.2.1 Denoising

Compared with the *in vivo* cough sound collected during tracheal intubation, body surface acquisition has the advantages of convenience and speed, with simple operation methods, low collection equipment requirements, and relaxed environmental requirements. However, due to its exposure to the open environment, there may be noise signals unrelated to cough sounds during the recording process, such as voices generated by patients and others during daily activities, talking voices during conversations, and environmental background noise, which may, in turn, cause interference in the recognition of cough sounds.

The production of a cough consists of three phases, namely, inhalation, pressurization, and exhalation, and the cough sound can be similarly divided into three phases: the initial, intermediate, and ending phases. The initial phase is the burst of coughing at the beginning, which is shorter in duration but rapidly increasing in energy. The middle phase is relatively smooth, when it is longer in duration and decreases in energy. The ending phase is the second burst before the end, which represents the periodic vibration of the vocal folds, when the energy is weaker than in the initial phase and which does not occur each time.

The frequency range of cough sounds is widely distributed, which is as low as 30 Hz and as high as 4,000 Hz. The frequency distribution of cough sounds is relatively uniform in its frequency range. In this paper, the patient’s self-recorded audio is used, and there will be noise such as environmental noise and speech, so the noise reduction process is carried out on the collected audio under the premise of retaining effective information on cough sound. The wavelet thresholding method is used for filtering and denoising by separating the useful information from the energy of the noise. Since the useful information is concentrated in the larger wavelet coefficients and the noise is mostly distributed in the smaller coefficients, filtering can be realized by removing the noise distributed in the smaller coefficients by setting a suitable threshold.

First, wavelet decomposition of one-dimensional signal is performed, the sym8 wavelet is selected, the wavelet decomposition level is 3, and the decomposition coefficients of each layer of the signal are calculated. A threshold is set for each layer coefficient, the signal above the threshold is retained, and the wavelet coefficients below the threshold are set to 0. Finally, wavelet reconstruction is performed, and the signal is recovered according to the low-frequency coefficients and high-frequency coefficients of each layer, and the denoised audio is obtained.

#### 2.2.2 Segmentation

Due to the long time of the collected audio data (more than 10 min), in order to ensure the computational speed of the subsequent segment extraction, feature recognition, and other processes, it is necessary to first carry out a coarse segmentation of the coughing sound, which is divided into a 20-s segment of short audio by time, and pad zeros at the end of data which is less than 20 s to ensure that each segment of the data after segmentation is 20 s.

A dual-threshold endpoint detection algorithm based on short-time energy and a short-time zero crossing rate is used for the segmented short audio data. Since the speech signal is generally divided into a silent segment, a clear segment, and a turbid segment, the silent segment is mainly background noise with the lowest average energy; the turbid segment is the corresponding speech signal segment emitted by the vibration of the vocal cords and has the highest average energy; the clear segment is the speech signal segment emitted by the friction, impact, or bursting of the air in the oral cavity and has an average energy between the two. By calculating the short-time energy and short-time zero crossing rate of the speech signal in each frame, the preliminary extracted segments are obtained by judging whether the speech segments are voiceless segments, clear-tone segments, or turbid-tone segments. Then, according to the duration and explosive characteristics of the cough segment, the restriction conditions of segment duration and maximum short-time energy are increased to preliminarily screen the segments that meet the characteristics of the cough sound and remove the wrongly extracted non-cough segments.

#### 2.2.3 Feature extraction

Since the segments extracted by the double threshold method may be false cough segments such as impact sound and ambient noise that are misrecognized, it is necessary to judge the potential cough segments after the endpoints are extracted. The candidate eigenvalues proposed to be used in this algorithm are mean amplitude, short-time zero crossing rate, short-time energy, Mel frequency cepstrum coefficient, and linear predictive cepstrum coefficient. After calculating the results of the above eigenvalues for some of the segments to be recognized, correlation analysis is carried out for each of these features according to the manually labeled labels of each segment, and a combination of the eigenvalues with higher correlation is determined for subsequent classification and recognition.

The average amplitude is the average value of signal’s amplitude over a period of time, and in this paper, the average amplitude of the extracted potential cough segments is calculated to reflect the magnitude of the sound intensity within the segment, which is calculated by the formula 1.

$$\left|\bar{x}\right|=\frac{1}{N}\sum_{i=1}^{N}\left|x_{i}\right|.$$ (1)

The short-time zero crossing rate is the number of times the signal crosses the zero value per frame of speech, which can reflect the frequency information on the signal (Bachu et al., 2010; Taquee et al., 2021), and its calculation formula is shown in Formula 2. Compared with other speech signals, cough sounds are characterized by a significant increase in the short-time zero crossing rate within the whole speech segment and larger than the zero crossing rate of general sound signals, so they can be used as a feature parameter for recognition.

$$Z_{n}=\frac{1}{2}\sum_{m=0}^{n-1}\left|sgn\left[x_{n}\left(m\right)\right]-sgn\left[x_{n}\left(m-1\right)\right]\right|.$$ (2)

The short-time energy (Formula 3) reflects the energy size of the speech signal in each frame, which can describe the change in the speech signal in the time domain and the strength of its bursting ability (Taquee et al., 2021). For cough tones, the strength of the explosive power of the signal reflects the intensity of cough, and in the initial explosive phase, due to the sudden increase in the volume of sound so that the short-time energy will have a significant rise, and its rise will be significantly higher than that of the cough tones and therefore can be used to distinguish between general sound signals and cough tones.

$$E_{n}=\sum_{m=-\infty }^{\infty }{\left[x\left(m\right)w\left(n-m\right)\right]}^{2}.$$ (3)

When $w\left(n\right)$ is a rectangular window and N is the frame length, the above equation can be expressed as Formula 4.

$$E_{n}=\sum_{m=0}^{N-1}{\left[x\left(m\right)\right]}^{2}.$$ (4)

Mel frequency cepstrum coefficients (MFCCs) are based on the results of human hearing experiments to analyze the spectrum of speech, describing the characteristics of the envelope (Duckitt et al., 2006). According to the conversion formula of Mel frequency to actual frequency $F_{mel}=1125\log\left(1+\frac{f}{700}\right)$ , the extraction of parametric features is performed by combining many frequency groups divided by the basilar membrane of the human ear as a Mel filter bank. The Mel filter bank is a triangular filter bank composed of M filters with a center frequency of $f\left(m\right)$ , which are equal-bandwidth in the Mel frequency range. The transfer function of each filter is as formula 5.

$$H_{m}\left(k\right)=\left\{\begin{array}{cc} 0 & k<f\left(m-1\right)\\ \frac{k-f\left(m-1\right)}{f\left(m\right)-f\left(m-1\right)} & f\left(m-1\right)\leq k\leq f\left(m\right)\\ \frac{f\left(m+1\right)-k}{f\left(m+1\right)-f\left(m\right)} & f\left(m\right)<k\leq f\left(m+1\right)\\ 0 & k>f\left(m+1\right) \end{array}\right.\;0\leq m\leq M,$$ (5)

where $f\left(m\right)=\left(\frac{N}{f_{s}}\right)F_{mel}^{-1}\left(F_{mel}\left(f_{l}\right)+m\frac{F_{mel}\left(f_{h}\right)-F_{mel}\left(f_{1}\right)}{M+1}\right)$ .

The FFT transform is performed on each frame of the signal to calculate the spectral line energy, which is then passed through the Mel filter, and the Mel inverse spectral coefficients are obtained by calculating the DCT after taking the logarithm of the Mel filter’s energy $S\;\left(i,m\right)$ , which is shown is Formula 6.

$$mfcc\left(i,n\right)=\sqrt{\frac{2}{M}}\sum_{m=0}^{M-1}\log\left[S\left(i,m\right)\right]\cos\left[\frac{\pi n\left(2m-1\right)}{2M}\right].$$ (6)

The linear predictive cepstrum coefficient (LPCC, which is calculated by Formula 7) is obtained by doing a Fourier inverse transform of the frequency response $H\left(e^{j\omega }\right)$ after logarithmically obtaining $\left.\left.\log\right|H\left(e^{j\omega }\right)\right|$ (Mammone et al., 1996). Since $H\left(e^{j\omega }\right)$ reflects the frequency response of the sound channel and the spectral envelope of the analyzed signal, the LPCC is also considered to contain the envelope information on the signal spectrum, which can be viewed as an approximation of the short-time inverse spectrum of the original signal. Linear prediction coefficients are generally utilized to obtain the LPCC, which is computationally small and easy to implement.

$$\left\{\begin{array}{c} \hat{h}\left(1\right)=a_{1}\\ \hat{h}\left(n\right)=a_{n}+\sum_{i=1}^{n-1}\left(1-\frac{i}{n}\right)a_{i}\hat{h}\left(n-i\right)\;1<n\leq p\\ \hat{h}\left(n\right)=\sum_{i=1}^{p}\left(1-\frac{i}{n}\right)a_{i}\hat{h}\left(n-i\right)\;n>p \end{array}\right..$$ (7)

#### 2.2.4 Recognition

Support vector machine is a binary classification model that achieves classification by finding an optimal decision boundary among many instances, which is suitable for dealing with classification tasks on small and medium-sized complex datasets (Sharan et al., 2019). In speech system recognition, its recognition results are better than the hidden Markov model, and it runs faster and has better generalization ability in the case of small sample datasets, so SVM is used for classification.

**FIGURE 2:**
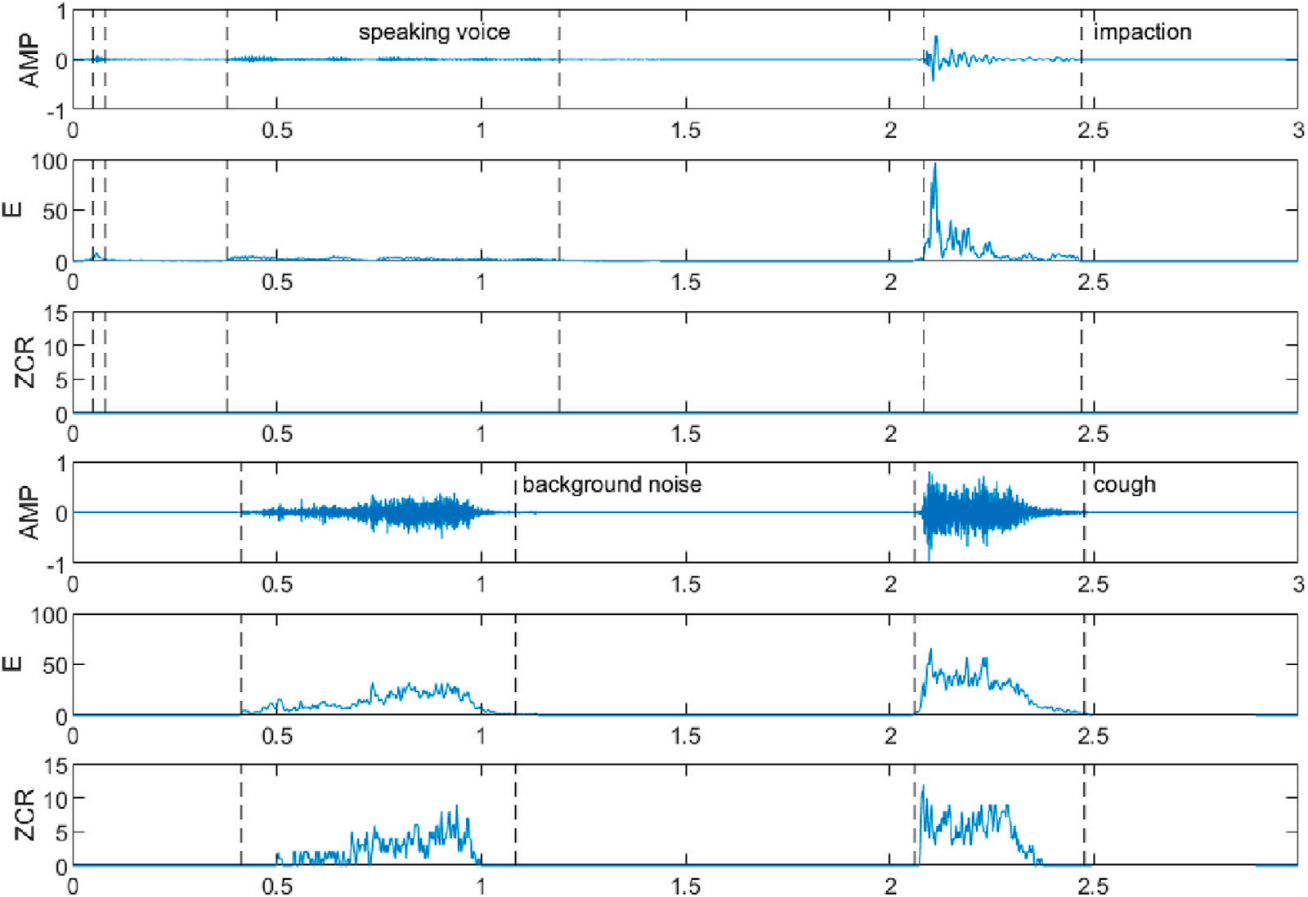
Waveforms, short-time energies, and short-time zero crossing rates of speaking voice, impaction, background noise, and cough fragments.

The double threshold method can only distinguish the voiceless, clear, and turbid sounds in a segment of speech and cannot distinguish the specific categories of sounds in the voiced segment, such as speaking, sneezing, impact, vibration, and background noise, and they have some differences in the above characteristics, among which the waveforms, short-time energies, and short-time over-zero rates of the speaking sound, the impact, the background noise, and the coughing sound are shown in Figure 2. It can be seen that compared with other sounds, the amplitude, short-time energy, and short-time zero crossing rate of coughing sound are higher, and the trend of its change is to increase rapidly to a larger value first and then slowly decrease. The amplitude, short-time energy, and short-time zero crossing rate of the speaking sound are lower; the impact sound only has higher amplitude and short-time energy, and the trend of short-time energy is similar to that of the cough sound, while the short-time zero crossing rate is basically 0; the amplitude, short-time energy, and short-time zero crossing rate of the background noise are all gradually increasing from low, and the degree of change in its short-time energy is relatively smooth.

Ten 20-s short audio segments (500 segments in total) from each patient were randomly selected and subjected to filtering, cough segment extraction, and other operations to obtain 977 speech segments including cough tone sounds and noises (speech, sneezes, crashes, vibrations, background noises, etc.). According to the feature value calculation process in 3), mean amplitude (M), mean short-time zero crossing rate, maximum short-time zero crossing rate within the segment (ZCR), mean short-time energy, maximum short-time energy within the segment (E), 12-dimensional mean Mel frequency cepstrum coefficients (MFCC), and 13-dimensional linear predictive cepstrum coefficients (LPCC) are calculated as the to-be-selected feature values and are labeled “Cough” or “Noise” according to the audio label content, forming the label vector of the dataset.

In order to reduce the amount of computation in the actual algorithm, the above to-be-selected eigenvalues are analyzed for correlation, different combinations of eigenvalues are selected for the training of the classifier, and combinations with higher accuracy are selected to form the final eigenvalue matrix.

#### 2.2.5 Counting

Define cough frequency as the number of coughs in 1 min, which can be calculated by Formula 8 since the duration of each segment of coarse segmented audio is 20 s.

$$F_{C}=\sum_{3}^{i=1}N_{i}.$$ (8)

### 2.3 System design

**FIGURE 3:**
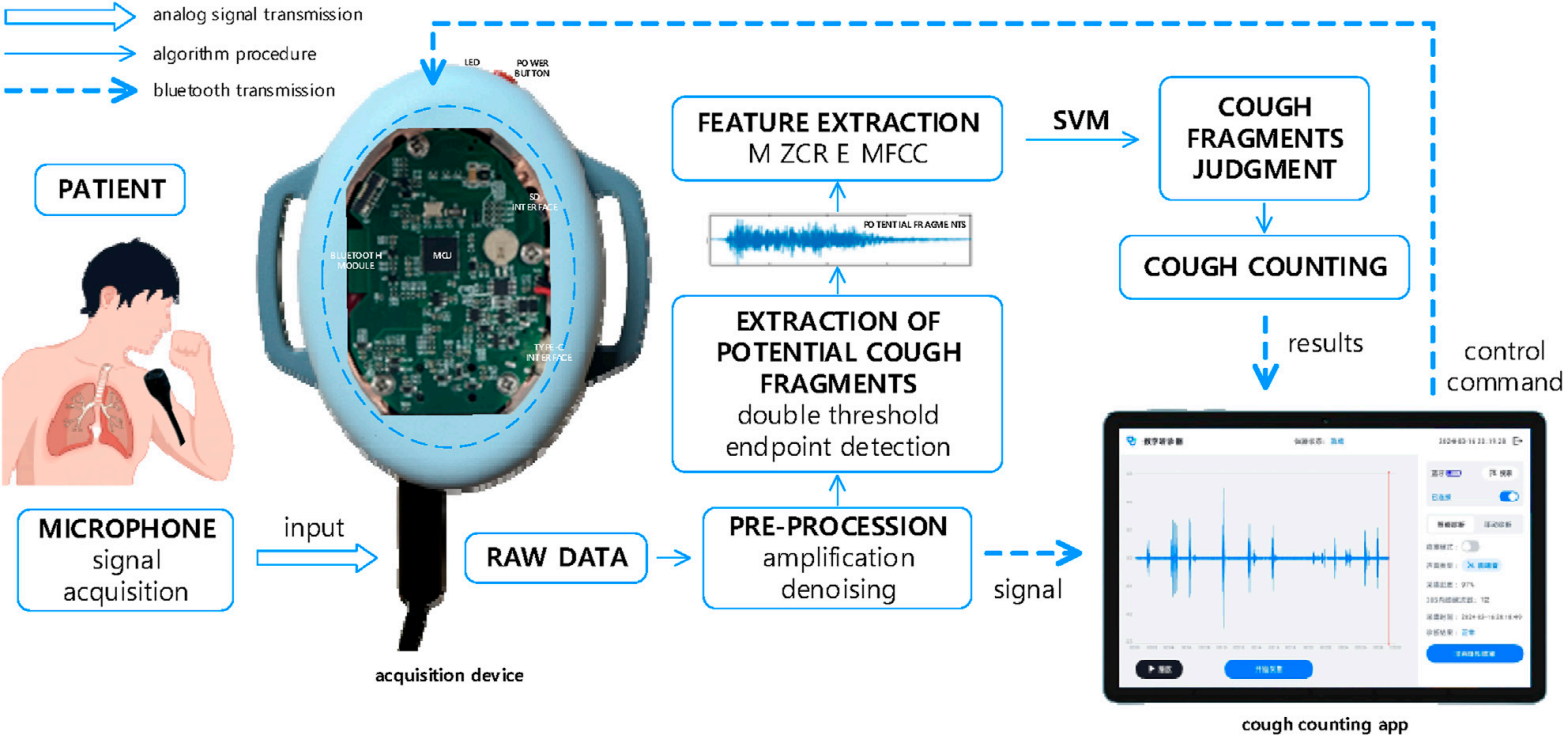
Block diagram of the wearable automatic cough counting system. The microphone is secured to the collar near the patient’s mouth, and the device can be secured to the patient’s waist or upper arm with a strap.

The system includes a wearable acquisition device and a receiving host computer, and the above counting algorithm is built into the acquisition device; the system block diagram is shown in Figure 3.

The wearable acquisition device using the STM32 series chip as the main control chip completes the system control processes such as acquisition, counting, data sending, and receiving. The data acquisition part of the 3.5-mm microphone interface can be connected to the AKG C417 lavalier mini microphone. The weight and volume of the microphone is small and does not give a foreign body feeling when wearing, so this can be maximized close to the source of sound acquisition. The Bluetooth transmission module is the HC-04 Bluetooth serial communication module; the working frequency band is 2.4 GHz ISM; using GFSK modulation, Bluetooth transmission can be realized within 10 m. The power module adopts a 1,200-mAh rechargeable lithium battery with a rated power of 4.4 Wh and output voltage of 3.7 V, and is equipped with a type-c port as the charging interface. The device adopts SD card for data storage, buzzer, and LED light as on/off and power and Bluetooth connection status indication. The device can be secured to the arm, waist, etc. or placed in a backpack by attaching it to an elastic band. The microphone is secured to clothing by a collar clip.

The receiving upper computer is a Huawei HONOR Pad X8 tablet with a built-in automatic cough counting APP, which is capable of realizing data transmission with the acquisition device via Bluetooth and displaying and playing back the acquired data and counting results. Since the acquisition device only has a power button, the APP can remotely control the device, including controlling the start of acquisition and setting the acquisition mode (normal mode or calibration mode).

## 3 Results

### 3.1 Filtering result

**FIGURE 4:**
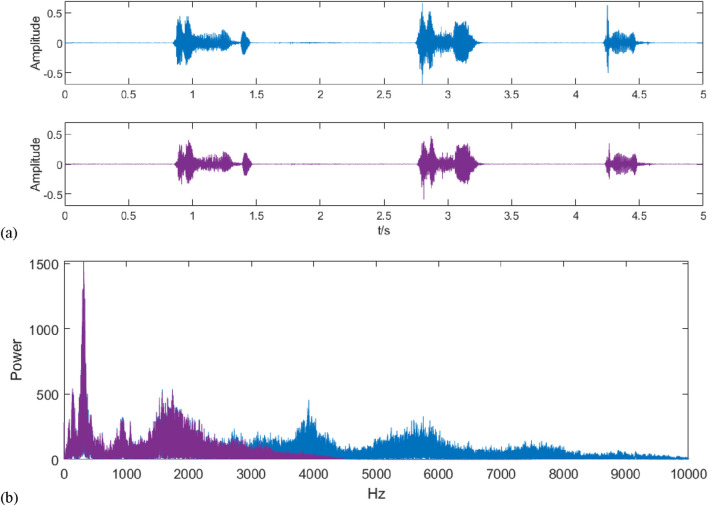
Signal waveform and spectrum after denoising. **(A)** shows the waveforms of the signals before and after filtering by wavelet transform, where blue represents the signal before filtering and purple represents the signal after filtering; **(B)** shows the spectrograms of the signals before and after filtering, where blue represents the signal before filtering and purple represents the signal after filtering, and it can be seen by comparison that the denoising process filters out the high-frequency noises above 3,000 Hz.

The original and filtered audio signals are shown in Figure 4A, and both are analyzed spectrally, as shown in Figure 4B. It can be seen that after wavelet denoising, the high-frequency interference present in the original signal is filtered out and the frequency band contains the frequency range in which the cough tone exists.

By calculating its average signal-to-noise ratio and comparing it, the average signal-to-noise ratio of 50 raw audio data is −28.4134, which is improved to 8.038 after filtering by the wavelet thresholding method.

### 3.2 Segmentation result

**FIGURE 5:**
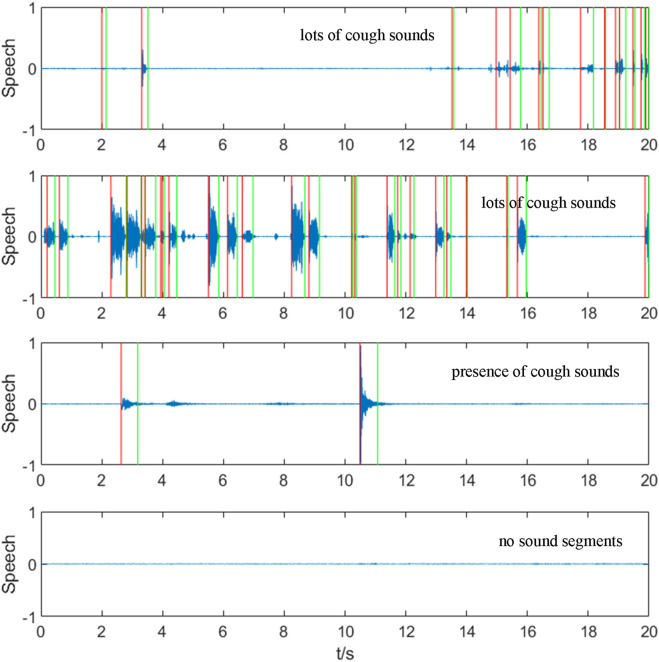
Segmentation results for different audio segments. The figure shows the segment extraction results for four random segments (lots of cough sounds, presence of cough sounds and interference, and no sound segments), with the start and end of potential cough segments identified using the double-threshold method marked by red and green lines.

The results of cough segment extraction based on the double-threshold method are shown in Figure 5, which demonstrates the segment extraction results of several random segments from different audios.

It can be seen that the double-threshold method with extra threshold limits is able to extract most of the cough segments, exclude as many non-cough segments as possible, and there is no misrecognition of long-time voiceless segments, which improves the specificity of the initial extraction for cough sounds and reduces the number of segments, for which the feature values need to be calculated subsequently. However, there are still some abnormally identified short silent segments (generally noise in the background environment), and it is not possible to distinguish cough segments from non-cough segments in the audible segments (speaking, sneezing, crashing, vibrating, and wheezing at the end of coughing), so the extracted segments need to be further analyzed with the help of other eigenvalues and SVM classifiers.

### 3.3 Counting result

**FIGURE 6:**
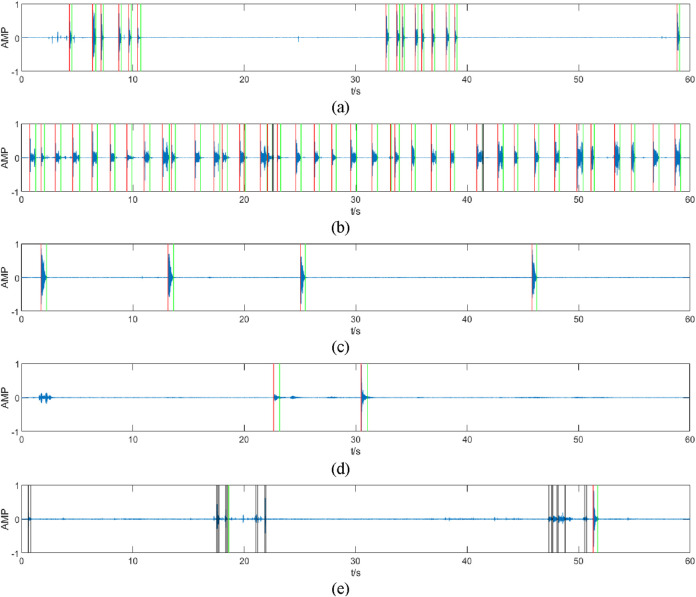
Results of different audio counts (partial). The figure **(A–E)** shows the recognition results of 5 different audios, the segments marked by red and green lines are those judged by the algorithm to be coughs, and the segments marked by black lines are those judged by the algorithm to be non-coughs. The recognition algorithm accurately recognizes the start and end points of coughs [in **(A,C)**] and removes mis-extracted segments such as coughing endings (which is very short and may cause double counting) **(B)**, noises **(E)**, and speaking sounds **(D)**.

The partial counting results for the different audios are shown in Figure 6, where the red line represents the starting point of the segments recognized as coughs, the green line represents the endpoint of the segments recognized as coughs, and the black line represents the segments that were extracted but not judged to be coughs.

Through the above processing flow, most of the cough segments can be extracted by the first double-threshold method, but contain some misclassified noise segments, and finally, the other sound segments that are mistakenly extracted can be removed after recognition by the SVM classifier.

## 4 Discussion

### 4.1 Verification of accuracy

The accuracy (ACC, which is calculated by Formula 9), sensitivity (SEN, which is calculated by Formula 10), specificity (SPE, which is calculated by Formula 11), Matthew correlation coefficient (MCC, which is calculated by Formula 12), positive predictive value (PPV, which is calculated by Formula 13), negative predictive value (NPV, which is calculated by Formula 14), and F1 score (which is calculated by Formula 15) are calculated for the different combinations with the following formula.

$$ACC=\frac{TP+TN}{TP+FN+TN+FP},$$ (9)

$$SEN=\frac{TP}{TP+FN},$$ (10)

$$SPE=\frac{TN}{TN+FP},$$ (11)

$$MCC=\frac{TP\times TN-FP\times FN}{\sqrt{\left(TP+FP\right)\left(TP+FN\right)\left(TN+FP\right)\left(TN+FN\right)}},$$ (12)

$$PPV=\frac{TP}{TP+FP},$$ (13)

$$NPV=\frac{TN}{TN+FN},$$ (14)

$$F_{1}=2\times \frac{\frac{TP}{TP+FP}\times \frac{TP}{TP+FN}}{\frac{TP}{TP+FP}+\frac{TP}{TP+FN}},$$ (15)

where TP is the true case, FN is the false negative case, FP is the false positive case, and TN is the true negative case.

It can be seen from Table 1 that the method using ① M + ZCR + E + MFCC as the feature matrix has the best recognition effect, with an accuracy of 93.24% and a correct recognition rate of 97.58% for coughs, and its Kappa value is calculated to be 0.9209, which is a good system consistency. Compared with ② M + ZCR + E + LPCC group, the correlation between actual classification and predicted classification is strong, and the classification performance is better; compared with ③ M + ZCR + E + MFCC + LPCC group, the classification performance of both of them is close to each other, but ① adopts the eigenvalue matrix with a lower dimension, which reduces the computational complexity.

**TABLE 1:** Classification results under different combinations of eigenvalues.

Combination	ACC (%)	SEN (%)	SPE (%)	MCC (%)	PPV (%)	NPV (%)	F1 score (%)
M + ZCR + E + MFCC	93.24	97.58	86.97	86.10	91.56	96.12	94.47
M + ZCR + E + LPCC	90.58	95.67	83.21	80.52	89.19	93.00	92.32
M + ZCR + E + MFCC + LPCC	91.91	97.58	83.71	83.44	89.67	95.98	93.45

### 4.2 Comparison with previous work

Comparing our approach with previous works (Monge-Alvarez et al., 2019; You et al., 2017; Sharan et al., 2019; Matos et al., 2006), as shown in Table 2, used the hidden Markov model to automatically detect cough sounds from continuous dynamic recordings, computed 39 MFCC features of cough events from nine patients, and rescaled the parameters by inverse spectral boosting, which yielded an accuracy of 82% and an error of seven coughs per hour. You et al. (2017) extracted unique spectral features of cough segments based on NMF and parameterized the spectral structure of coughs using multiple Gaussian distributions and scaling parameters and used SVM for classification, obtaining an accuracy of 81.5%. Monge-Alvarez et al. (2019) proposed an audio-based robust cough segmentation for the machine hearing system to compute the set of short-time spectral features for different frequency bands of cough segments of 13 patients under different levels of noise environments and selected the short-time features that were not affected by the environment as the feature matrix of the SVM, which achieved a sensitivity of 83.31% and a specificity of 79.26%. Sharan et al. (2019) used cochlear spectrogram and linear MFCC for feature extraction to automatically segment the cough recordings of 479 patients with various clinical diagnoses of respiratory tract infections, and SVM was used to recognize coughs in the extracted segments, achieving an accuracy of 86.09%, a sensitivity of 92.31%, and a specificity of 85.29%.

**TABLE 2:** Comparison of others’ work.

Literature	ACC (%)	SEN (%)	SPE (%)	F1 score (%)
HMM + MFCC Matos et al. (2006)	82	—	—	—
SVM + NMF-G You et al. (2017)	81.50	84.40	85.40	80.70
SVM + short-time feature Monge-Alvarez et al. (2019)	—	83.31	79.26	—
SVM + MFCC-CIF Sharan et al. (2019)	86.09	92.31	85.29	59.99
Our approach	93.24	97.58	86.97	94.47

Comparing the above methods, the method of this paper has better results in 1) recognition effect, 2) feature dimension, 3) data authenticity, and 4) practical applicability. 1) The method of this paper performs the best in terms of accuracy, sensitivity, and specificity compared to the literature (Monge-Alvarez et al., 2019; You et al., 2017; Sharan et al., 2019; Matos et al., 2006) with more than 90% accuracy, and it has the highest sensitivity, which is 5.27% more than that of Sharan et al. (2019) which indicates a better recognition rate of cough segments. Although the detection of non-cough segments is poorer, it still has a good performance compared to the rest of the methods. 2) The literature (Monge-Alvarez et al., 2019; You et al., 2017; Matos et al., 2006) uses 39, 60, and 29 dimensional features, respectively. In this paper, we use a lower number of dimensions (17 dimensions) to achieve a high accuracy, and the calculation method is simple, with a good correlation (>0.4) and high significance (*p* < 0.01), which reduces the pre-computational complexity. 3) The previous methods mostly used cough segments collected by volunteers in the laboratory or manually divided (Monge-Alvarez et al., 2019; You et al., 2017; Matos et al., 2006); although different noise environments were simulated (Monge-Alvarez et al., 2019) or volunteers were made to simulate life scenarios, they still differed from the process of cough detection carried out in real scenarios. In this paper, we used the data captured in the patients’ real life (in which the sound of other people communicating, keyboard sounds at work, and other sounds occurring in life can be heard), which is highly authentic. 4) The previous methods pay more attention to judging the manually segmented cough segments and lack the automatic extraction of the complete audio, and the literature (Sharan et al., 2019) designed a method to automatically segment the cough segments, but its accuracy and specificity are poor after combining with a classifier. This paper carries out a complete processing flow (preprocessing, segmentation, feature extraction, classification and identification, and counting) for the original audio that has been actually collected and not been processed, without manually marking the segments. It can automatically identify the segments to be tested and then divide them, realizing the complete detection process, which has a good practical application value.

### 4.3 Recognition effect

This method improves the relevance of the traditional double-threshold method for the recognition of cough segments and reduces the subsequent computational complexity by the extra threshold value; according to the characteristics of the cough segments, it constructs feature values with a better correlation with the cough segments and completes the differentiation between the cough segments and the non-cough segments through the SVM classifier to realize the cough count within 1 min.

The algorithm has high accuracy, high sensitivity, low complexity, good counting effect, and small error from the actual counting value, which can be applied to small portable devices with poor computing power. The average counting error for 60-s length audio is 0.46 times, and the average running time is 2.80 ± 2.27 s, compared with the algorithm that requires 0.506 s of computing time for the 1-s audio segment (Shin et al., 2009), which can generate counting results in a timely manner, and has both strong real-time performance and can be used for real-time cough counting. The recognition effect of partial samples is shown in Table 3.

**TABLE 3:** Mean counting error over 60 s for partial samples and running times for 20- and 60-s speech segments.

Sample no.	Average counting error/60 s (time)	Running time (20 s) (s)	Running time (60 s) (s)
17	0.3	0.72 ± 0.49	4.85 ± 2.59
19	0.1	0.61 ± 0.32	2.09 ± 0.73
30	0.7	0.61 ± 0.34	3.42 ± 1.70
47	0.2	0.50 ± 0.21	2.91 ± 1.16
48	0.9	0.15 ± 0.03	0.75 ± 0.21

### 4.4 Limitations

As the patient may be recorded in the hospital ward, the presence of other patients with similar conditions around them may lead to different people’s coughs being recorded in, resulting in inaccurate recognition of the number of coughs, which can be achieved by judging the distance between the sound source and the recording device according to the volume level of the cough segment, thus distinguishing between the patient’s own cough and the interfering cough. When the device is in a strong noise environment, cough sounds cannot be clearly recorded; the user can be informed whether the current environment is suitable for monitoring by assessing the current ambient noise level.

When the cough sound appears to overlap with other sounds or due to the presence of a loud interference source near the recording device (e.g., cell phone vibration on the desktop), it is not possible to identify the clip as a cough segment at this point, and this problem is similar to the cough events that existed in Amrulloh et al. (2015) that refused to be voice-corrupted, so the segment can be assumed to have been voice-corrupted, and thus, it can be judged as a non-cough segment.

This method ignores the effect of truncation of audio segment boundaries and does not take into account the problem that coughs are disconnected when they appear between two audio segments, leading to segmentation, and thus cannot be recognized or recognized repeatedly. In the future, a 2–5-s overlapping segment will be added to the part between two audio segments, to reduce the effect of the truncation of the speech boundaries on the counting of cough sounds.

## 5 Conclusion

In this paper, an automatic cough counting method is proposed to present a complete set of the cough counting process based on real captured audio data, including noise reduction, segment extraction, eigenvalue calculation, recognition, and counting process. The accuracy, sensitivity, specificity, and F1 score of the algorithm are 93.24%, 97.58%, 86.97%, and 94.47%, respectively, and the Kappa value is 0.9209, which is in good agreement.

The algorithm overcomes the problem of different resolution of different recording devices and reduces the recording requirements for the capture device to facilitate the capture in life. The sensitivity of the double threshold method to cough segments is improved in terms of threshold and eigenvalues, and the computational speed is improved so that real-time and accuracy can be taken into account, and the effectiveness of the algorithm is verified in real application environments by real non-contact cell phone recordings. The feature value dimension is low, and the computational complexity of each feature is low, which realizes a better detection effect under low dimension. The complete processing flow of real audio realizes the automatic counting of audio, which has a strong application ability and adaptability to the real scene.

In addition, a wearable automatic cough counting system was designed based on the present algorithm, which is small, convenient, and easy to wear, and combined with the accompanying application software program, it is capable of realizing long-time real-time monitoring of patients’ coughs inside and outside the hospital. Patients wear the device for a long time without strong foreign body sensation, and the device has no major impact on the user’s daily life.

In the future, the number of coughs can be combined with the intensity of coughing, the nature of coughing, and the peak time period to diagnose the severity of the patient’s condition and to realize a more comprehensive judgment and early warning of the patient’s condition through real-time monitoring throughout the day. By fixing this system on the upper arm of the patient with respiratory disease, after collecting the data by a microphone, the patient’s coughing frequency within 1 h is analyzed, and the monitoring platform can be connected to upload the data to the nurses’ station to complete the automatic monitoring of the patient’s coughing frequency.

## Data Availability

The raw data supporting the conclusions of this article will be made available by the authors, without undue reservation.
